# Stress Situations and Speech Fluency: A Pilot Study of Oral Presentations in Immersive Virtual Reality Environments

**DOI:** 10.3390/bs15121652

**Published:** 2025-12-01

**Authors:** Yasna Sandoval, Bárbara Farías, Luis Gajardo, Soledad Quezada Cáceres, Gabriel Lagos, Carlos Rojas

**Affiliations:** 1 Department of Health Rehabilitation Sciences, University of Bío-Bío, Chillán 3780000, Chile; ysandoval@ubiobio.cl (Y.S.); bfarias@ubiobio.cl (B.F.); glagos@ubiobio.cl (G.L.); 2 Department of Computer Science and Information Technology, University of Bío-Bío, Chillán 3780000, Chile; lgajardo@ubiobio.cl; 3 Department of Visual Communication, University of Bío-Bío, Chillán 3780000, Chile; soquezada@ubiobio.cl

**Keywords:** speech fluency, stress situations, virtual reality, oral exposure, virtual environment

## Abstract

This pilot study investigates the relationship between stress situations and speech fluency in virtual reality environments. It aims to analyze how different stress scenarios, classified into low-, medium-, and high-stress environments, can affect speech rate, increase syllable/word repetitions, and lead to hesitations in university students. Previous research has established connections between stress situations and speech fluency, highlighting that stress can negatively influence behavior, cognitive processes, and communicative performance across various contexts, including oral presentations. An experiment was conducted with 30 participants randomly divided into three groups. Each group was exposed to different virtual stress environments (low/medium/high) during simulated oral presentations. A virtual reality platform was created to establish controlled environments and monitor the participants’ fluency in real time. An Analysis of Variance (ANOVA) test revealed that participants in the low-stress virtual environment performed better, achieving higher word and syllable production. In contrast, the high-stress virtual environment demonstrated an increase in disfluencies and hesitations. Results emphasize the impact of stress situations on oral communication, advocating for the use of virtual reality technology as a means of preparing individuals for challenging speaking scenarios. This approach has the potential to enhance speech fluency as a result of targeted practice in stress-inducing environments; that is to say, alleviating anxiety and improving overall communicative efficacy.

## 1. Introduction

Speech fluency is defined as the ability to produce continuous, clear, coherent, and uninterrupted speech, making it a fundamental skill for oral communication (Sandoval et al., 2022). This ability is not only an indicator of linguistic competence, but also of the speaker’s executive, cognitive and emotional skills (Guitar, 2019). On the other hand, oral presentations are necessary tools for personal and professional development, where fluency plays a key role. Delivering a good oral presentation facilitates effective communication and positively influences perceptions of an individual’s performance and confidence in academic or work environments (Dunbar et al., 2006).

Research on speech fluency has shown that environmental, biological, cognitive and emotional factors of the individual can influence this ability. For example, one of the factors having an impact on fluency is stress (Yan, 2019). In this context, virtual reality (VR) offers an immersive and interactive platform, allowing the simulation of stress situations in oral presentation scenarios (i.e., a work meeting) using a methodology that cannot be replicated by traditional tools (Olszewski et al., 2016; Lara et al., 2019). VR creates realistic and controlled environments that allow researchers to manipulate key variables such as audience size, feedback, and presentation settings, being this an unmatched capability with traditional methodologies. Studies indicate that exposure to VR can provoke physiological stress responses similar to those encountered in actual public speaking scenarios, enabling a deeper understanding of how individuals manage anxiety in these contexts (Schröder & Mühlberger, 2023; Martens et al., 2019). Furthermore, VR technology can incorporate biometric feedback systems for real-time monitoring of physiological responses, offering a comprehensive evaluation of stress reactions during simulated presentations. Additionally, VR environments also replicate various social dynamics reflective of real-life interactions, allowing participants to practice and address their stress in a safe and immersive context (Kroczek & Mühlberger, 2023).

According to Jackson et al. (2016), the stress experienced by speakers in different situations could significantly affect their oral performance. For example, high-pressure VR scenarios (e.g., an end-of-year job speech) coupled with specific stressful situations (e.g., a phone ringing during a presentation) would further increase the individual’s stress, which could hypothetically be reflected in reduced fluency. In addition, stress activates physiological responses in the body that could hinder the articulation and cohesion of ideas, thus exacerbating speech production blockages (Peeters, 2019).

### 1.1. Stress Situations and Speech Fluency

Stress is defined as a state of physical or mental exhaustion resulting from a negative interaction between the person and his or her environment. This phenomenon occurs when environmental demands exceed the individual’s capacity to respond, creating an imbalance that can trigger adverse physiological and emotional responses (Cohen et al., 2016). As a result, stress situations could affect behavior, thoughts and communicative performance in various activities, such as oral presentations. Similarly, stress situations are significantly associated with the presence of distracting elements in the environment, such as unexpected noises or situations, exacerbating the effects of stress and interfering with verbal memory and word production (Arrivillaga et al., 2020). As a result, speakers focus on their stress and less on the content they want to communicate, leading to errors such as hesitations and repetitions (Serna, 2023).

The effect of stress situations on different speech parameters, such as speech rate, syllable repetition, word repetition and hesitations, has been documented in several investigations (Gómez, 2018; Kappen et al., 2022). For example, stress situations could significantly alter the rate of speech production, which is often measured in terms of syllables per minute or words per minute (de Oliveira & Furquim, 2008; Guitar, 2019). In stressful situations, speakers tend to show a slower rate of speech due to increased cognitive load, tension and discomfort. Pisanski et al. (2016) suggest that physiological responses to stress may modulate speech characteristics, including rate. Thus, stress and distracting situations create a negative cycle that exacerbates a person’s fluency and communicative efficiency.

In addition, stress situations could increase the repetition of syllables and words during speech, leading to the presence of disfluencies. During periods of high stress, people repeat more syllables or words as a coping mechanism, demonstrating a “struggle” to maintain speech continuity without interruptions (Buchanan et al., 2014). In addition, the cognitive demands associated with delivering a high-stress oral presentation could affect memory retrieval, making it difficult to access the lexical system (Rojas et al., 2022) and leading to increased syllable or word repetitions (Yan, 2019).

Hesitations—long pauses or the use of filler words such as ‘eh’ or ‘well’—would also be common in speech under stress situations. Research has found that adolescent speakers show increased markers of hesitation when faced with stress in speech tasks (Gokgoz-Kurt, 2023). These interruptions would not only affect fluency, but also affect listener perception, as insecurity or lack of knowledge might be perceived (Gokgoz-Kurt, 2023). Baird et al. (2019) mention that speech produced in stress-induced scenarios often shows less fluency and an increase in interruptions due to physiological arousal, which complicates speech articulation (Contreras et al., 2020), thus highlighting the importance of designing challenging virtual environments to train users in high-stress situations.

### 1.2. Virtual Reality and Stress Scenarios

The concept of “virtual reality” was introduced in 1965 by Ivan Sutherland, who published a seminal article that laid the foundation for the term (Wohlgenannt et al., 2020). Over time, the equipment associated with VR systems has gradually evolved, leading to their active adoption in a wide range of fields. In the clinical context, their use has been explored to efficiently educate, assess and train individuals (Kim et al., 2021). This immersive capability allows participants to submerge themselves in situations that are difficult to replicate in real life, fostering interactions that increase both immersion and user engagement (Lara et al., 2019).

Mel Slater has made important contribution on how immersive VR can induce stress and anxiety during oral presentations, specifically public speaking, particularly through the induction of presence—the sensation of truly “being there” in a virtual setting—have been foundational. Slater et al. (2009) defined the concepts of place illusion and plausibility illusion, illustrating why individuals experience genuine stress when presenting to virtual audiences. His earlier work in 2006 (Slater et al., 2006) involved measuring real stress responses by employing physiological sensors—such as heart rate, skin conductance, and brain activity—to demonstrate that VR social scenarios, like addressing a virtual crowd, provoke authentic anxiety responses analogous to those faced in real-life situations. Further enhancing the scientific rigor of VR, Slater et al. (2010) established methods to objectively quantify presence, moving beyond reliance on subjective questionnaires. This validates the notion that even simplistic virtual audiences can elicit notable public speaking anxiety, particularly when participants receive negative feedback.

The design of the virtual stage also plays a crucial role in how stress manifests. For instance, spaces that simulate large audiences and unexpected situations, such as interruptions from ringing mobile phones, people coughing, etc., could increase participants’ sense of stress, having a negative impact on speech fluency, increasing blocking, hesitation and extension (Glémarec et al., 2021). According to Davydov et al. (2005), at a physiological level, perceived stress levels are associated with an increase in hormones such as epinephrine, which would have direct effects on the cognitive and motor functions necessary for speech production (Kim et al., 2021).

VR-based intervention programs have been shown to be effective in speech therapy and stress management in public presentation situations (Brock, 2023; Huang et al., 2020; Notaro et al., 2021; Tentu et al., 2024), allowing participants to experience and learn to manage their emotional reactivity in a controlled environment (Díaz-Pereira et al., 2025; Thrasher, 2022). This approach not only provides a safe space for users to practice and improve their communication skills, but also allows them to face the same circumstances they would in real life (Tan et al., 2025). Immersion in a virtual environment facilitates learning and practice by providing more authentic experiences than traditional forms of training, such as classroom practice or the use of more conventional methods (Kryston et al., 2021; Peng et al., 2018).

In addition, the ability to evaluate the participant’s speech more objectively is one of the key advantages of VR. The incorporation of biofeedback techniques allows not only fluency but also other physiological responses to stress to be monitored (Kudo et al., 2014). This combination improves the quality of learning, as it allows tailoring interventions to different levels of stress situations and provides a framework for implementing personalized strategies that optimize communicative competence (García et al., 2012). All in all, VR is a valuable resource for exploring stressful situations and training fluency in them. It enables participants to confront challenging scenarios in a secure and managed setting, enhancing persuasion and charisma while alleviating anxiety (Valls-Ratés et al., 2023). This fosters an environment conducive to learning and personal development.

### 1.3. The Present Study

This article addresses an important area at the intersection of stress situations and speech fluency in VR environments. Speech fluency is a fundamental component of effective communication, so it is important to understand how stress situations may affect this ability during oral exposures. Recent publications have shown that interruptions or unusual situations during oral presentations (e.g., external noise or conversations) can increase the physiological stress experienced by speakers (Battambang University Research Team, 2025; Li et al., 2025; Starr, 2025; Sörqvist et al., 2024). This study aims to analyze variations in speech rate, extensions, syllable repetition, and hesitation frequency experienced by participants under different levels of stress situations (low, medium and high) in a controlled virtual environment. Considering the innovative nature of the technological proposal applied to speech and behavioral sciences, particularly concerning stress situations, a controlled pilot study will be conducted. This pilot study will serve as a foundational assessment, designed to evaluate the effectiveness of the VR environments created for simulating stress in oral presentations. By using advanced VR technology, we can procure an authentic representation of real-world stressors that individuals face when performing public speaking tasks (Brundage et al., 2006; Díaz-Pereira et al., 2025; Satake et al., 2023). By focusing on how VR can simulate stressful situations in a controlled manner, it is anticipated that the results of this research will not only enrich knowledge about the inherent challenges associated with communication and, in particular, public speaking skills, but will also provide an initial empirical basis for the development of practices aimed at improving expository skills in diverse populations.

## 2. Materials and Methods

### 2.1. Participants

A sample of 30 third-grade Speech and Language Therapy students from the sponsoring university, who received a bonus of one credit for their elective courses, was selected by interest. Participants were aged between 19 and 21 years and included both males and females. Specific eligibility criteria were defined; inclusion criteria required participants to be university students with visual and hearing impairments that could be compensated by the use of glasses and/or hearing aids. On the other hand, participants who self-reported speech or voice disorders, cardiovascular disease, balance disorders, or diagnoses such as depression or neurological disorders were excluded from the study. Participants who reported high levels of academic stress (according to the survey used) were also excluded. To ensure the integrity of the findings and maintain a tightly controlled study group, a pilot study that systematically selects participants without existing conditions has been carried out. By refining participant selection to those without heightened academic stress or other significant impairments, this pilot study will allow for a more focused examination of stress effects on speech fluency in VR environments. Furthermore, to ensure the participants’ understanding of the nature of the study and its implications, as well as their voluntary participation, an informed consent form approved by the scientific ethics committee of the sponsoring university was read and signed by the subjects (code cecubb2024/1).

The 30 participants who made up the sample were randomly divided into three groups of ten, so that each group was exposed to different levels of stress (low, medium, high). The participants had to give an individual oral presentation (on a specific topic) in a VR scenario with different stress situations. For the oral presentation, the participants had a time limit of 120 s. Participants in group 1 were exposed to a low level of stress (scenario 1), as they only faced two stressful situations during their oral presentation. The situations were presented in the 20th and 40th second of the presentation. In contrast, participants in Group 2 were exposed to a medium level of stress, as they were faced with five stressful situations presented in the 10th, 30th, 50th, 70th and 90th second of the presentation (scenario 2). Finally, group 3 was exposed to a high level of stress. In this scenario they were presented with eight stressful situations, which appeared at seconds 5, 20, 35, 50, 65, 80, 95 and 110 (scenario 3).

The stress scenarios were defined in levels from lower to higher, as there is evidence that repeated exposure to stressful situations generates more stress due to the accumulation of fatigue that compromises the individual’s physiological resources. This phenomenon is explained by the overloading of regulatory systems and the decrease in resilience, creating a vicious cycle in the stress response (McEwen, 2004; McEwen & Gianaros, 2010). Therefore, and in line with some studies that claim that interruptions during oral presentations increase the speaker’s stress (Battambang University Research Team, 2025; Li et al., 2025; Starr, 2025; Sörqvist et al., 2024), the more stressful situations the participants were presented with, the more stress they could accumulate. For more details on the stress situations and scenarios created, see Table 1.

### 2.2. Materials and Design

#### 2.2.1. Platform Generation—Web and Virtual Reality Components

Prior to the experimental phase, a remote platform was created to store data, generate the desired VR scenarios and record the results, among other functions. The first component generated was the World Wide Web (web), which in its implementation had two subcomponents: the backend and the frontend, which interacted closely through REST-type services, thus favoring flexibility, scalability and better system performance. The backend sub-component consisted of the database and the code that represented the logic of the web application, using MySQL 8.0 technology for its high performance and scalability, particularly suitable for smaller projects. The web application was implemented using the Django framework (version 4.2) together with the Python 3.10 programming language, ensuring a modular and robust architecture, structured according to the Model-View-Template (MVT) pattern, which facilitated the integration of future functionalities based on artificial intelligence. The front-end was responsible for data visualization in the web browser, presenting visual interfaces to the user using technologies such as HTML, CSS and JavaScript.

**Figure 1:**
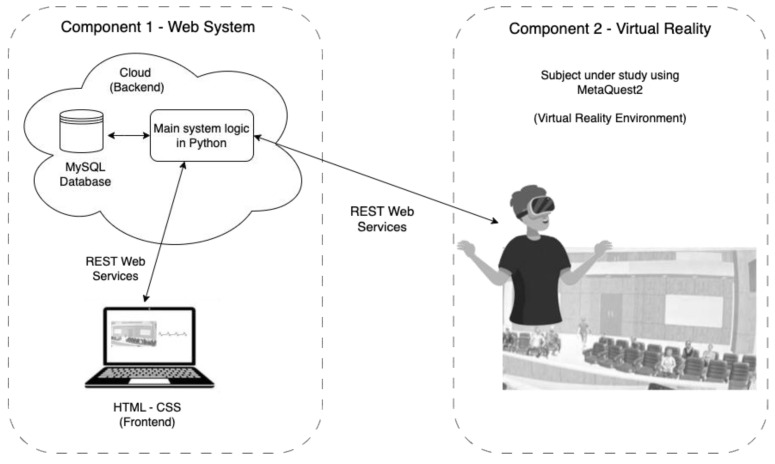
Interaction between components of the developed system.

The second component of the platform was the VR application, which had to provide an experience that simulated oral presentations in a highly immersive environment, allowing students to practice their speeches in realistic conditions, with different levels of distraction and pressure. Key features included the simulation of a virtual audience that reacted in different ways to the speaker’s speech, and the modulation of external stress-generating elements such as ambient noise or audience interruptions. This component allowed us to configure various aspects of the exhibition scenario, such as audience size, lighting and distractors, creating a controlled environment that could be adapted to different situations. From the web interface, the exhibitor visualized a three-dimensional environment, while the collected data, including audio recordings, were transmitted to the web platform for further analysis. In this way, the combination of these technologies not only provided an immersive experience, but also facilitated the assessment of students’ fluency in a realistic pressure context. The implementation of this component was carried out using the Unity VR platform, using the C# programming language and optimized for use in the Oculus Quest 2 viewer (to understand the interaction between the components, see Figure 1).

#### 2.2.2. Generation of the Topic for Oral Presentation

The experiment required all participants to deliver an impromptu oral presentation on the same topic. Subjects were informed two days prior about the topic to be presented. As a result, a current, general-interest text was produced providing all participants with a baseline of knowledge on the subject.

The structure of the text was as follows: (1) A short survey was carried out among the students in order to define the specific topic to be dealt with. The topic with the highest frequency of occurrence (use of social networks) was chosen. (2) Relevant information on the topic was collected through grey literature (magazines, newspapers or websites of interest). (3) Two language teachers, independent of this research, using the collected material, prepared the final text. (4) The text underwent a thorough review and editing process by the research team to rectify any grammatical and content-related errors. This diligent scrutiny ensured that the information presented is both accurate and original. For access to the revised text, refer to Supplementary Material S1.

### 2.3. Procedure

It consisted of three stages. In the first stage, the researcher responsible met with all second- and third-year subjects at the sponsoring university to invite them to participate in the research. General objectives were explained. A schedule was drawn up for the registered participants. Sessions were set to be individual and confidential. The day prior to the evaluation, subjects received the previously prepared information text “Influence of social networks in university life”. Additionally, attire advice –comfortable clothing- was given so they could live the VR experience.

The second stage consisted of a personal interview with the participants. This took place in a box in the Language, Speech and Cognition Laboratory of the sponsoring university and lasted 15 min per participant. The interview consisted of: (1) a medical history was taken to establish eligibility criteria; (2) participants were perceptually assessed for speech or voice disorders; (3) the Academic Stress Guideline (see Supplementary Material S2) was applied; (4) the procedures to be performed were explained and confidentiality was requested; (5) participants were asked to read and sign the informed consent form. Participants who met the eligibility criteria and signed the consent form proceeded to phase 3 of the study.

#### Experimental Procedure

**Figure 2:**
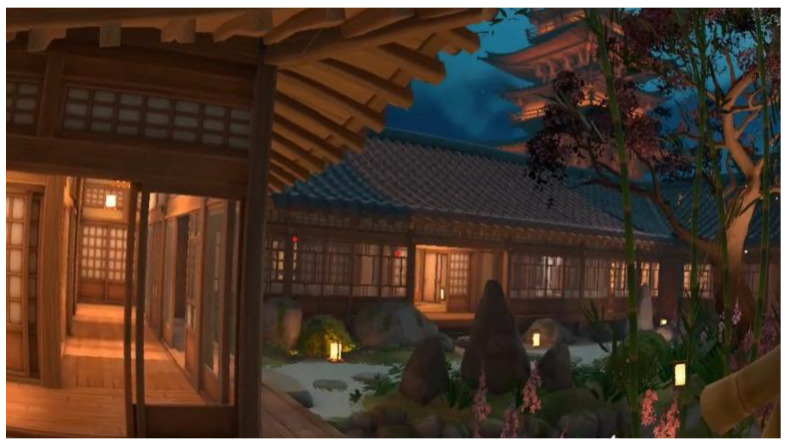
Initial relaxing VR environment (Japanese garden with soothing music).

The experimental procedure (Stage 3) was carried out after the interview and lasted approximately 10 min per participant. The evaluation took place in the VR laboratory of the sponsoring institution. First, the participant’s ID was registered in the web platform and a scenario was randomly assigned (low/medium/high stress). The VR headset was then installed to ensure the participant’s comfort. They were shown the default settings of the software without being introduced to any visual scenario, and were also instructed on the use of commands, buttons and scrolling in the virtual environment. To initiate the VR experience, all participants were immersed in a Japanese garden-like environment (see Figure 2), without any external stressful situation. They were instructed to simply observe their surroundings and enjoy the music.

**Figure 3:**
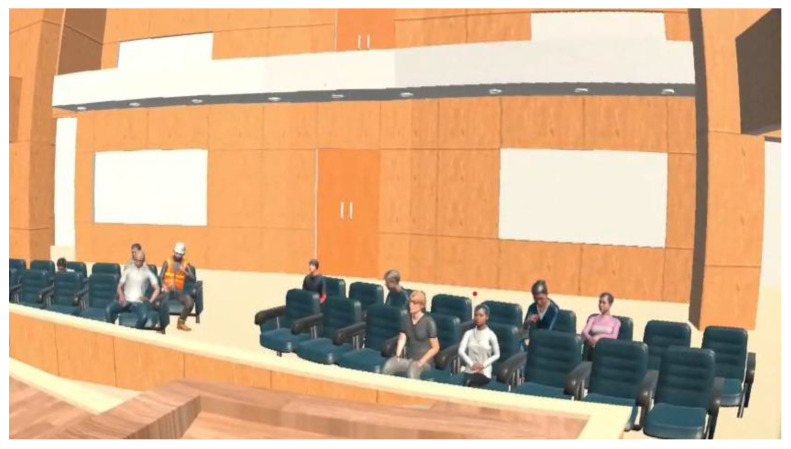
VR environment of oral presentation (Auditorium of the sponsoring university).

Once the Japanese garden environment was closed, the participants were instructed to prepare an oral presentation on the topic previously analyzed (the influence of social networks on university life), in which they were asked to give a lecture on the content explicit in the text and to express their opinions on the topic. As mentioned above, the three scenarios presented corresponded to the auditorium of the sponsoring university (the scenarios differed only in the number of stress situations presented), so it was a virtual scenario familiar to the participants, which could generate a high level of stress for them (see Figure 3). Beginning, the specific instruction was: “OK, if you’re ready, we can go. You have time, so take it easy.” At this point, the stage was opened and the participant could proceed. In case of prolonged silence during the presentation, the evaluator could give feedback only once, with the following instructions: *“what else can you say”, “give more details” “what is your opinion about it” “relax, tell me more, there is still time”.* Their oral intervention was recorded using the built-in microphone of the Meta Quest 2 Oculus headset (Reality Labs, Menlo Park, CA, USA) and VR software.

Once the two-minute presentation was over and the stage was closed, participants were asked how they felt and what they thought of the activity in general. The headset and equipment were removed. Following the completion of their presentations, students were required to complete a structured satisfaction survey comprising five dichotomous (‘yes/no’) questions designed to capture their overall experience (to view the survey and its results, please check Supplementary Material S3). The purpose of this survey was to assess various dimensions of their engagement in the VR environment, including enjoyment, ease of navigation, perceived realism, quality of guidance provided, and willingness to recommend the platform to peers.

### 2.4. Data Analysis

The analysis of speech fluency in this study adheres to the criteria defined by Guitar (2019). Specifically, the first 200 words were extracted from speech samples and several fluency parameters were measured: total words, total syllables, words per minute (Totwords × min), syllables per minute (Tot-sil × min), extensions, hesitations and syllable repetitions. This analysis was conducted using a systematic procedure aimed at ensuring accuracy and repeatability. First, a thorough auditory review was conducted of each participant’s oral presentation recordings. Second, the first 200 words spoken by each participant were transcribed meticulously. Third, the total number of words and syllables were counted. Then, incidences of extensions, hesitations, and syllable repetitions were systematically recorded. Finally, the processing rate was calculated in terms of words per minute, and the articulatory rate in terms of syllables per minute. The data were stored using Microsoft Excel, version 2024.

Descriptive statistics were used for each of the study variables. A non-parametric inferential analysis was then performed using ANOVA (analysis of variance) in Python 3.12 (2024) online software. The ANOVA test is a statistical tool commonly used to compare means between three or more groups. However, its use is limited by several assumptions, the most critical being normality of the data and homogeneity of variances. When working with small samples (as in the present pilot study) and there is evidence that the data do not meet the assumptions of normality, it is critical to consider nonparametric ANOVAs. A major justification for using a nonparametric approach is that it is less dependent on assumptions about the distribution of the data. However, studies have shown that although ANOVA can be relatively robust to violations of normality, its statistical power may suffer (Kikvidze & Moya-Laraño, 2008).

Upon identifying significant overall differences through ANOVA, a follow-up procedure involving post hoc tests is essential for clarifying specific group differences. Bonferroni correction is a post hoc test adjustment used in statistical analysis to counteract the problem of multiple comparisons. When performing multiple hypothesis tests simultaneously (e.g., comparing several groups in ANOVA), the chance of obtaining a false positive (Type I error) increases. The Bonferroni correction controls the family-wise error rate (FWER) by dividing the desired overall alpha level (usually 0.05) by the number of comparisons being made.

## 3. Results

The tables below show the mean, standard deviation, mode, minimum, maximum and significant values for each fluency variable and in each of the scenarios. The sample consisted of 30 people with an average age of 21, 24 females and 6 males. In general, the tables show variations between the scenarios, resulting in noticeable changes in word and syllable production, as well as in disfluencies, extensions and hesitations. These results not only illustrate the influence of stress situations on oral presentations, but also highlight important implications for the design of virtual presentation environments that support communicative fluency.

As for scenario 1, which included 2 stress situations, it showed an adequate level of performance in terms of fluency. Although the number of extensions and hesitations was moderate, the overall production of words and syllables suggested a reasonable level of confidence among the participants. The context of exposure to only 2 stress situations seems to favor comfort, as most participants presented a minimal number of syllable repetitions and hesitations (see Table 2).

Table 3 shows the results of scenario 2 (5 stress situations). Similarly to scenario 1, adequate oral production is observed, but the multiplied number of extensions and hesitations suggests an increase in the participants’ nervousness or stress before the different situations presented. This could indicate that although the participants are able to generate large amounts of speech, the context causes disfluencies that affect the fluency of their speech.

Scenario 3, on the other hand, which consisted of 8 stressful situations, showed a more limited oral production performance compared to the previous ones, which could be associated with a higher level of stress or difficulties in presenting in a less familiar or more tiring environment. The lower rates of verbal production, together with the high number of syllables, suggest that although the participants managed to maintain the conversation, they faced significant challenges which were reflected in greater extensions and hesitations (see Table 4).

Comparing the results of the three scenarios, trends in verbal production and disfluencies were observed. Scenario 1 stood out with the highest fluency and production, being the most favorable. Scenario 2 also had high production, but with more disfluencies, which could be related to greater nervousness. Finally, Scenario 3 showed that the participants were challenged in terms of fluency, with more hesitations and extensions. This analysis highlights how the conditions of each scenario influence verbal performance (and presumably communication strategies), and suggests that a controlled environment may facilitate natural expression, while a less familiar and more tiring environment increases disfluencies.

The comparisons between groups are shown below. These were determined using a nonparametric ANOVA statistical test, followed by a Bonferroni post hoc test to correct for multiple comparisons and identify significant differences between scenarios based on the calculated variables (see Table 5).

Analysis of the results showed that Scenario 1 had a higher average word (198.4) and syllable (244.0) production than the other two scenarios, suggesting a more favorable environment for fluency. Differences in the rate of words per minute were also significant, with an average of 134.2 in Scenario 1 compared to 74.2 in Scenario 3, where the lowest performance was recorded (*p* < 0.05). In addition, extensions and hesitations were more pronounced in the third scenario (25 and 1.2), suggesting that participants may have experienced higher levels of stress, leading to greater difficulties in fluency.

On the other hand, the data from scenario 2 showed a reasonable average number of words (199.3) and syllables (238.9), but with a high presence of extensions (9.2) and hesitations (2.4), suggesting that although participants maintained a high level of verbal production, contextual pressures may have caused significant disfluencies. Further comparison of the mean scores across the three scenarios revealed that scenario 3 was characterized by a higher number of extensions (25.0) and lower word and syllable production, explaining the variation in overall scores (*p* < 0.05). These results illustrate the direct influence of the presentation context on participants’ verbal fluency and justify the need to analyses and adapt exposure environments in order to optimize communicative expression in academic and professional situations.

## 4. Discussion

The interaction between stress situations and speech fluency during oral presentations in VR environments is an important area of research in fields such as psychology, linguistics and education. It has been documented that stress-induced anxiety could affect an individual’s communicative performance, making their speech less coherent and less fluent (Grieve et al., 2021). Findings from previous research highlight the negative effect that stress situations could have on speech fluency during oral presentations, emphasizing that stress can interact with multiple variables such as speech content and the context of the presentation environment (Battambang University Research Team, 2025; Li et al., 2025; Starr, 2025; Sörqvist et al., 2024). In particular, these studies show that different levels of stress situations significantly affect different parameters of verbal performance, such as total words produced and fluency rates (Dwyer & Davidson, 2021). For instance, a study by Grieve et al. (2021) found that students who experienced high levels of oral presentation anxiety tended to avoid public speaking situations, which impacted on their communicative development. The obtained data indicate that participants performed best in scenario 1, where stress was minimal, reaching an average production of 198.4 words. These results are consistent with previous research which suggests that lower levels of stress correlate with improved speech quality and fluency, providing a clear context for the importance of a relaxed environment for effective speech.

On the other hand, Scenario 3 demonstrated the challenges associated with increased levels of stress situations, reflected in a significantly lower average total number of words produced (178.8) and a worrying increase in extensions (25.0). This increase in stress-related disfluency and nervousness is consistent with the findings of studies conducted by Kappen et al. (2022), who noted that intense stress can lead to a decrease in both speech rate and fluency due to cognitive overload. Other studies have suggested that stress situations could lead to an internal struggle between the individual’s desire to communicate effectively and the emotional pressure they feel, affecting their ability to articulate clearly (Davis et al., 2020). Interestingly, additional research supports the claim that stress can cause a cognitive load that limits the speaker’s ability to access their lexicon and articulate their thoughts (Díaz-Pereira et al., 2025; Satake et al., 2023).

In this matter, it has been documented that high levels of stress could lead to increase anxiety and nervousness, resulting in a reduced rate of speech and increased hesitations. In the context of the current study, participants who experienced elevated levels of stress (scenario 3) showed lower average word production, accompanied by a marked increase in syllable extensions and hesitations. For example, an average of 25.0 extensions reflects how emotional baggage can hinder fluent speech, as stress can act as a significant distractor. Research suggests that in situations of high anxiety, individuals tend to divert their attention to their own fears and worries, which hinders their ability to access the lexicon and evidences hesitations in their verbal production (Chard & van Zalk, 2022; Peeters, 2019). Thus, stress detection and management becomes critical to facilitate more fluent and effective speech in oral presentations, especially in contexts where VR adds an additional layer of complexity and pressure.

It is noteworthy to mention an unusual finding: given the stressful conditions of the oral presentation, the mean number of extensions in Scenarios 1 and 3 (21 and 25, respectively) can be considered normal. Surprisingly, however, Scenario 2 yielded a significantly lower number of extensions (mean = 9.2) than Scenario 1. While a definitive explanation is not possible, we hypothesize that this difference stems from the specific composition of Group 2, which included several confident speakers who were less susceptible to stress and thus produced fewer extensions. Supporting this view, the highest individual score in Group 2 was 28 extensions, compared to 36 in Group 1 and 47 in Group 3.

The use of VR as a platform for this study provides a critical framework to analyze the dynamic interaction between stress situations and speech. VR provides a uniquely immersive experience that can replicate high-stress situations, allowing for a deeper understanding of how individuals react under stress. In this regard, research has shown that VR can increase the perception of realism in simulated situations, which is relevant as more realistic environments could exacerbate the stress response and negatively affect communication. For example, Peeters (2019) reports that simulating stressful scenarios in VR could induce emotional and physiological responses comparable to those observed in real-life situations. Similarly, Lim et al. (2023) concluded that VR environments designed to induce stress could affect communicative effectiveness and create an increased sense of fatigue in users. This phenomenon has been observed in several studies documenting an increase in hesitations and extensions of speech, confirming that the associated emotional load can affect fluency (Lim et al., 2023).

The results of Scenario 2 support this notion, as participants showed a marked increase in hesitations (2.4). This increase in disfluency is supported by literature suggesting that highly stressful simulated environments activate physiological responses that may negatively impact speech fluency. Previous research has emphasized that stress activates the fight or flight response, which may lead to increased physiological arousal and thus affect articulation and speech coordination (Peeters, 2019).

On the other hand, the use of VR in oral fluency training offers innovative potential to mitigate the negative effects of stress. VR allows users to practice in controlled environments that simulate real-life situations in which oral discourse is required. This immersion helps to familiarize participants with the dynamics of the situation and can desensitize them to the stressful situations associated with public speaking. According to previous studies, VR has been shown to be effective in reducing stress and improving speaker confidence, resulting in greater fluency during speech (Lim et al., 2023). This is consistent with research that has explored how practice in virtual environments could reduce anxiety and improves verbal performance, contributing not only to the development of communicative skills but also to the emotional well-being of users (Díaz-Pereira et al., 2025; Kryston et al., 2021; Rizzo et al., 2010; Satake et al., 2023).

In terms of familiarity with the technological environment, this is an additional factor that could influence participants’ experience of stressful situations. When confronted with new and potentially complex technology, users may experience uncertainty, which could increase their stress during oral presentations. Some studies suggest that preparation and familiarity with the tools and environment could help to reduce stress and improve communicative performance (Amanvermez et al., 2023; Rama et al., 2023).

The research highlights the potential of VR as a platform for intervention in communication skills under stress situations. Given the positive correlation between stress management techniques and improved performance (Amanvermez et al., 2023; Rama et al., 2023), incorporating elements of familiarization with VR technology could significantly improve participants’ preparedness in stressful situations. Therefore, it is imperative that future research explores how varying levels of familiarity with VR technology and the implementation of specific stress management techniques affect speech fluency outcomes. Programs that use VR to simulate oral presentations and allow for repeated practice could be effective in reducing stress and improving speaker confidence, resulting in more fluent and effective speech. Education and speech therapy could also benefit greatly from this approach. As documented in previous studies, exposure to simulated situations would allow participants to experience the process of public speaking in a safe and controlled environment (Jiménez, 2018; As & Setiawan, 2021).

### Limitations and Future Perspectives

Despite the significant contributions of this pilot study, it is important to acknowledge its limitations. The small sample size of 30 participants restricts the generalizability of the findings, as the insights gathered may not be fully representative of the broader population. Although pilot studies offer the advantage of allowing researchers to explore innovative technological applications in speech and behavioral sciences, they also come with limitations concerning the projection of results. Consequently, while the data provide preliminary insights into the relationship between stress and speech fluency in VR environments, the results should be interpreted cautiously, as broader conclusions may not readily apply without further investigation in more extensive studies.

Furthermore, not assessing stress using physiological tests was a limitation. Not having such limitation would have allowed for a better correlation of the study variables. Future studies should address this issue to determine the extent to which stress increases with an increased number of interruptions during oral speech.

To fully realize the potential implications of using VR for managing speech fluency amid stress, it is crucial to scale this study to a larger population. Future research should aim to include a more diverse demographic by increasing the sample size and ensuring varied participant backgrounds to enhance the external validity of the results. By conducting larger-scale studies, researchers can better assess the consistency and robustness of the effects noted in this pilot, as well as explore variations in outcomes as a function of factors such as age, gender, and previous experience with public speaking or VR technology. Ultimately, scaling up the study will provide a more comprehensive understanding of how to leverage VR to assist individuals in refining their communicative competencies under stress, thus contributing valuable insights to the fields of education and speech therapy.

## 5. Conclusions

In conclusion, the study has provided important findings regarding the relationship between stress situations and speech fluency during oral presentations in VR environments. The results indicate that fluency in verbal production could be affected by the level of stress induced in different situations. Specifically, participants demonstrated a good performance in the scenario with fewer stress stimuli, evidenced by high production of words and syllables, as well as a low incidence of disfluencies. This aligns with previous research that highlights how lower levels of stress situations correlate with improved communicative performance.

The proliferation in the number of extensions and hesitations in scenarios with higher stress levels suggests that environmental conditions could destabilize speakers’ abilities to communicate effectively. These findings emphasize the necessity of considering stress management in communication practices, particularly in presentation contexts that demand a high level of verbal performance. However, it is important to acknowledge the limitations of the study, primarily the small sample size and the inability to directly measure stress levels, which could restrict the generalizability of the results.

## Figures and Tables

**Table 1:** Stress situations presented in VR for each scenario.

* Stress Situation	Scenario 1	Scenario 2	Scenario 3
A person enters the auditorium by surprise.	✔	✔	✔
Loud cell phone ringing	✔	✔	✔
Person leaves the auditorium unexpectedly		✔	✔
Person raises hand and asks a question		✔	✔
Loud fire siren noise		✔	✔
Person coughs loudly			✔
Two people converse and interrupt			✔
Person points to the exhibitor			✔

* Each stress situation was presented consecutively; there was no overlapping between them.

**Table 2:** Speech fluency variables with 2 stress situations (Scenario 1).

Variable	Average	St. Dev.	Mode	Minimum	Maximum
Age	20.3	0.78	20	19	21
Total Words	198.4	29.36	198	156	270
Total Syllables	244.0	42.46	254	180	365
* Tot-words × min	134.2	25.68	187	106	187
** Tot-sil × min	135.8	31.29	120	102	183
Extensions	21.0	5.39	21	11	36
Hesitations	0.5	0.52	0	0	2
Syllable repetitions	0.1	0.32	0	0	1

* Total words per minute; ** Total syllables per minute.

**Table 3:** Speech fluency variables with 5 stress situations (Scenario 2).

Variable	Average	St. Dev.	Mode	Minimum	Maximum
Age	20.5	0.75	20	19	22
Total Words	199.3	34.58	165	139	277
Total Syllables	238.9	66.73	211	199	532
* Tot-words × min	103.2	25.88	89	59	187
** Tot-sil × min	145.9	50.95	123	67	243
Extensions	9.2	2.95	11	8	28
Hesitations	2.4	1.10	1	0	4
Syllable repetitions	0.3	0.47	0	0	1

* Total words per minute; ** Total syllables per minute.

**Table 4:** Speech fluency variables with 8 stress situations (Scenario 3).

Variable	Average	St. Dev.	Mode	Minimum	Maximum
Age	22.5	3.67	21	19	34
Total Words	178.8	25.14	172	119	215
Total Syllables	292.7	82.27	323	225	416
* Tot-words × min	74.2	15.22	63	52	93
** Tot-sil × min	126.6	24.75	116	86	183
Extensions	25.0	9.24	41	20	47
Hesitations	1.2	0.64	1	0	5
Syllable repetitions	0.4	0.52	0	0	4

* Total words per minute; ** Total syllables per minute.

**Table 5:** Comparison between VR scenarios. ANOVA between groups (Bonferroni’s post hoc test corrected).

Variable	Scenario	Average	St. Dev.	Min.	Max.	*p*-Value (1 vs. 2)	*p*-Value (1 vs. 3)	*p*-Value (2 vs. 3)
Total words	1	198.4	29.36	156	270	0.845	<0.001 ***	0.037 ***
	2	199.3	34.58	139	277			
	3	178.8	25.14	119	215			
Total syllables	1	244.0	42.46	180	365	0.845	0.015 ***	<0.001 ***
	2	238.9	66.73	199	532			
	3	292.7	82.27	225	416			
* Tot-words × min	1	134.2	25.68	106	187	0.845	<0.001 ***	0.045 ***
	2	103.2	25.88	59	187			
	3	74.2	15.22	52	93			
** Tot-sil × min	1	135.8	31.29	102	183	0.845	0.012 ***	0.034 ***
	2	145.9	50.95	67	243			
	3	126.6	24.75	86	183			
Extensions	1	21.0	5.39	11	36	0.845	0.091	0.002 ***
	2	9.2	2.95	8	28			
	3	25.0	9.24	20	47			
Hesitations	1	0.5	0.52	0	2	0.845	0.012 ***	0.022 ***
	2	2.4	1.10	0	4			
	3	1.2	0.64	0	5			
Syllable repetition	1	0.1	0.32	0	1	0.845	0.134	0.546
	2	0.3	0.47	0	1			
	3	0.4	0.52	0	4			

* Total words per minute; ** Total syllables per minute; *** *p* < 0.05.

## Data Availability

The raw data supporting the conclusions of this article will be made available by the authors on request.

## Supplementary Materials

The following supporting information can be downloaded at: https://www.mdpi.com/article/10.3390/bs15121652/s1, Supplementary Material S1 (Text generated for oral speech production); Supplementary Material S2 (Academic Stress Guideline) and Supplementary Material S3 (VR satisfaction survey).
