# Supplementary material for: Stress Situations and Speech Fluency: A Pilot Study of Oral Presentations in Immersive Virtual Reality Environments
Source: Behav Sci (Basel). 2025 Dec 1;15(12):1652. doi: 10.3390/bs15121652 (PMC12729271; doi:10.3390/bs15121652)
Supplement: Supplementary file 1 [file behavsci-15-01652-s001.zip › behavsci-3944436-supplementary.pdf]

Supplementary Material 1

Lasredessocialeshantransformadoprofundamentediversosaspectosdenuestrasvidas, incluyendoel ámbito acadimico. Enunmundocada vezmásconectado, estasplataformasnosolo influyenen cómonoscomunicamos, sinotambiinencómoaprendemos, compartimosconocimientosy nospreparamospara el futuro.

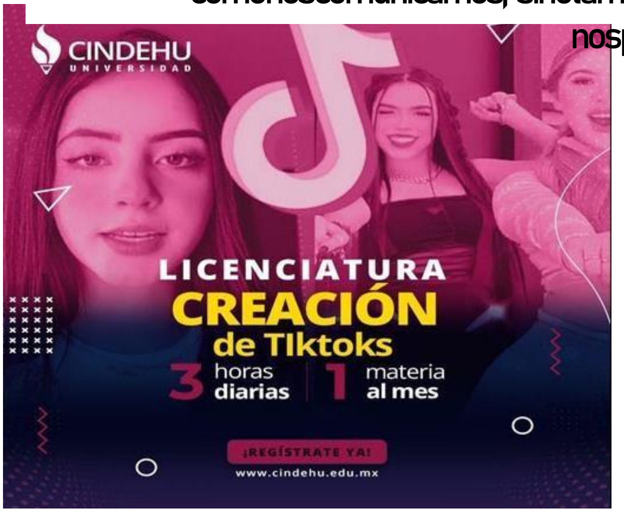

INFUENCIA DE LAS RRSS EN LA VIDA UNIVERSITARIA

¿Todo es tan malo?

Las redes sociales son una herramienta poderosa en la vida universitaria actual, capaz de enriquecer el proceso de aprendizaje, facilitar la colaboración y preparar a los estudiantes para el mundo profesional. Sin embargo, es esencial que se utilicen con moderación y conciencia para evitar sus posibles efectos negativos. Al final del día, la clave está en encontrar un equilibrio entre el aprovechamiento de sus beneficios y la gestión de sus desafíos.

REPORTAJE

SITIO WEB:
www.ubb.cl/noticias

CORREO ELECTRÓNICO:
ysandoval@ubiobio.cl

Acceso inmediato a la información

Una de las mayores influencias de las redes sociales en la vida universitaria es el acceso instantáneo a una vasta cantidad de información. Estudiantes y profesores pueden seguir cuentas dedicadas a temas específicos, desde matemáticas hasta literatura, y estar al día con las últimas investigaciones y tendencias. Además, plataformas como YouTube, Twitter y LinkedIn permiten acceder a contenido educativo de alta calidad de manera gratuita o a bajo costo, complementando los recursos tradicionales como libros y clases presenciales.

Aprendizaje colaborativo

Las redes sociales facilitan el aprendizaje colaborativo, un enfoque que ha ganado popularidad en el entorno educativo. A través de grupos de estudio en Facebook, chats de grupo en WhatsApp o hilos de discusión en Reddit, los estudiantes pueden intercambiar ideas, resolver dudas en tiempo real y trabajar en proyectos de manera conjunta, incluso si se encuentran en diferentes partes del mundo. Esta colaboración no solo mejora la comprensión de los temas, sino que también fomenta habilidades blandas como el trabajo en equipo y la comunicación efectiva.

Desarrollo de la marca personal y redes profesionales

Plataformas como LinkedIn han hecho que la creación de una red profesional comience mucho antes de graduarse. Los estudiantes pueden conectarse con profesionales del área que les interesa, seguir a empresas y líderes de opinión, e incluso compartir sus propios logros académicos y proyectos personales. Esto no solo les da una ventaja competitiva al ingresar al mercado laboral, sino que también les permite construir una marca personal desde temprano en su carrera.

Desafíos y distracciones

Sin embargo, no todo es positivo. El uso de redes sociales también plantea desafíos en el ámbito académico, especialmente en términos de distracción. Las notificaciones constantes, el contenido diseñado para captar la atención y la tendencia a procrastinar pueden afectar la concentración y la productividad de los estudiantes. Por ello, es crucial que los usuarios aprendan a gestionar su tiempo y utilicen estas plataformas de manera consciente y equilibrada.

El impacto en la salud mental

Por otro lado, la presión por mantener una imagen perfecta en redes como Instagram o TikTok puede generar ansiedad y estrés, especialmente entre los jóvenes. La comparación constante con los logros y la vida de los demás puede afectar la autoestima y, en consecuencia, el rendimiento académico. Por eso, es importante fomentar un uso saludable de las redes, enfocándose en su potencial educativo y no solo en su aspecto social.

University

Supplementary Material 2

Escala de evaluación del estrés en estudiantes universitarios

Nombre:

A continuación se presenta una pauta breve que incluye una serie de afirmaciones relacionadas con distintas dimensiones del estrés académico, donde los estudiantes pueden responder en una escala de 1 a 5, donde 1 representa "Totalmente en desacuerdo" y 5 "Totalmente de acuerdo". Por favor, indique su grado de acuerdo con las siguientes afirmaciones respecto a su experiencia académica actual.

Carga académica: Siento que tengo demasiadas tareas y deberes que realizar.

1 2 3 4 5

Exámenes y evaluaciones: Los exámenes me provocan ansiedad significativa.

1 2 3 4 5

Tiempo para realizar actividades: Me resulta difícil encontrar tiempo para actividades personales debido a mis estudios.

1 2 3 4 5

Relaciones sociales: Me siento aislado/a de mis compañeros debido a las exigencias académicas.

1 2 3 4 5

Afrontamiento: Me siento capaz de manejar efectivamente el estrés que proviene de mis estudios.

1 2 3 4 5

Categorías de interpretación de resultados:

1-5: Bajo nivel de estrés
6-10: Estrés leve
11-15: Estrés moderado
16-20: Estrés alto
21-25: Estrés muy alto

Supplementary Material 3

Table. Detailed VR Satisfaction Survey Results Summarized

Survey Question	Total (n=30)	Yes (%)	Comments/Notes
Did you enjoy your VR experience in the learning environment?	Yes: 27, No: 3	90,0 %	Majority enjoyed it; high engagement.
Did you find the VR environment easy to navigate?	Yes: 25, No: 5	83,0 %	Navigation was generally rated easy.
Did you feel that the VR technology provided a realistic learning experience?	Yes: 25, No: 5	83,0 %	Participants appreciated realism.
Did you feel that there was enough guidance provided for using the VR equipment?	Yes: 27, No: 3	90,0 %	Some desired more guidance.
Would you recommend VR learning experiences to other students?	Yes: 26, No: 4	87,0 %	High likelihood of recommendations.
